# Evaluation of prognostic models developed using standardised image features from different PET automated segmentation methods

**DOI:** 10.1186/s13550-018-0379-3

**Published:** 2018-04-11

**Authors:** Craig Parkinson, Kieran Foley, Philip Whybra, Robert Hills, Ashley Roberts, Chris Marshall, John Staffurth, Emiliano Spezi

**Affiliations:** 1 School of Engineering, Cardiff University, Queen’s Buildings, 14-17 The Parade, Cardiff, CF24 3AA UK; 2 Division of Cancer and Genetics, School of Medicine, UHW Main Building, Heath Park, Cardiff, CF14 4XN UK; 3 Clinical Trials Unit, Cardiff University, Cardiff, CF10 3AT UK; 4 Clinical Radiology, University Hospital of Wales, Heath Park, Cardiff, CF14 4XW UK; 5 Wales Research and Diagnostic PET Imaging Centre, Cardiff University, School of Medicine, Ground Floor, C Block, UHW Main Building, Heath Park, Cardiff, CF14 4XN UK; 6 Velindre Cancer Centre, Velindre Rd, Cardiff, CF14 2TL UK

**Keywords:** Prognostic model, Esophageal cancer, PET/CT, Automated segmentation

## Abstract

**Background:**

Prognosis in oesophageal cancer (OC) is poor. The 5-year overall survival (OS) rate is approximately 15%. Personalised medicine is hoped to increase the 5- and 10-year OS rates. Quantitative analysis of PET is gaining substantial interest in prognostic research but requires the accurate definition of the metabolic tumour volume. This study compares prognostic models developed in the same patient cohort using individual PET segmentation algorithms and assesses the impact on patient risk stratification.

Consecutive patients (*n* = 427) with biopsy-proven OC were included in final analysis. All patients were staged with PET/CT between September 2010 and July 2016. Nine automatic PET segmentation methods were studied. All tumour contours were subjectively analysed for accuracy, and segmentation methods with < 90% accuracy were excluded. Standardised image features were calculated, and a series of prognostic models were developed using identical clinical data. The proportion of patients changing risk classification group were calculated.

**Results:**

Out of nine PET segmentation methods studied, clustering means (KM2), general clustering means (GCM3), adaptive thresholding (AT) and watershed thresholding (WT) methods were included for analysis. Known clinical prognostic factors (age, treatment and staging) were significant in all of the developed prognostic models. AT and KM2 segmentation methods developed identical prognostic models. Patient risk stratification was dependent on the segmentation method used to develop the prognostic model with up to 73 patients (17.1%) changing risk stratification group.

**Conclusion:**

Prognostic models incorporating quantitative image features are dependent on the method used to delineate the primary tumour. This has a subsequent effect on risk stratification, with patients changing groups depending on the image segmentation method used.

**Electronic supplementary material:**

The online version of this article (10.1186/s13550-018-0379-3) contains supplementary material, which is available to authorized users.

## Highlights

- Texture features are dependent on the segmentation method
- Prognostic scores differ between models derived using different segmentation methods
- Patient risk stratification using identical clinical data is dependent on the segmentation method

## Background

Prognosis in oesophageal cancer (OC) is poor. The 1- and 5-year overall survival (OS) rate is 44 and 15%, respectively [1]. The aim of precision medicine and prognostic models is to ensure each patient is managed with the most appropriate treatment, which may improve patient OS [2–4]. The avoidance of futile aggressive therapies prevents unnecessary treatment and improves quality of life. In addition, better patient stratification may also allow more efficient trial designs.

Prognostic models are formulated from patient specific information such as age, pathological subtype, molecular characterisation and tumour staging. However, the advanced quantitative analysis of medical images, especially CT, MR and PET, is gaining substantial interest in prognostic research as more accurate prognostic models may be developed. Radiomic features characterise tumour phentotypes through extraction of high-dimensional data [5] and can be associated with metastatic growth, recurrence and survival in several solid cancers [6]. These methods may also have added prognostic value in cancer staging pathways [7].

The accurate delineation of the relevant metabolic tumour volume (MTV) on PET/CT is challenging due to low spatial resolution and the high noise characteristics of PET imaging [8]. Many different PET segmentation techniques have been proposed as a solution to the delineation of the MTV [9]. Numerous PET-based radiomic features have been described, but the results of radiomic analysis are highly dependent on the method used to derive the MTV [10]. Few studies have compared results of radiomic analysis derived from each segmentation method (cf. [11] and references therein) or have investigated their effect on patient risk stratification derived from prognostic models [12–14].

This study aimed to develop a series of prognostic models in the same patient cohort using identical clinical data and standardised radiomic features derived from different segmentation methods. The impact of using different segmentation methods on patient risk stratification was assessed.

## Methods

### Patient cohort

This is a retrospective cohort study of consecutive patients with biopsy-proven OC, including gastro-oesophageal junctional (GOJ) tumours, radiologically staged between 16 September 2010 and 31 July 2016. Patients were identified from a database of OC patients used in a previous study [15]. Institutional Review Board approval was granted and requirement for informed consent was waived (Wales REC 1, UK reference 14/WA/1208).

Overall, 486 patients with FDG-avid primary oesophageal and GOJ tumours were considered for inclusion. Fourteen patients were excluded due to missing clinical data. All patients were deemed to have potentially curable disease following contrast-enhanced CT staging investigation. All PET/CT examinations were performed separately, following the initial CT, and reported in the same centre by Consultant Radiologists with an interest in Nuclear Medicine. Radiological staging was performed according to the International Union Against Cancer (UICC) TNM 7th edition [16]. Following exclusions, 472 patients were studied.

### PET/CT protocol

Patients were fasted for at least 6 h prior to tracer administration. Serum glucose levels were routinely checked and confirmed as less than 7.0 mmol/L prior to imaging. Patients received a dose of 4 MBq of ^18^F-FDG/kg. Uptake time was 90 min, standard practice at our institution. A GE 690 scanner (GE Healthcare, Buckinghamshire, UK) was used. CT images were acquired in a helical acquisition with a pitch of 0.98 and tube rotation speed of 0.5 s. Tube output was 120 kVp with output modulation between 20 and 200 mA. Matrix size for the CT acquisition was 512 × 512 pixels with a 50 cm field of view. No oral or intravenous contrast was administered. PET images were acquired at 3 min per field of view. The length of the axial field of view was 15.7 cm (skull base to mid-thigh). Images were reconstructed with the ordered subset expectation maximisation algorithm, with 24 subsets and 2 iterations. Matrix size was 256 × 256 pixels, using the VUE Point™ time of flight algorithm. All PET-based data was obtained using the same PET/CT scanner and reconstruction method with voxel dimensions of 2.73 × 2.73 × 3.27 mm.

### Treatment protocols

Patients began treatment 2–4 weeks after staging FDG PET/CT imaging. Patients either had endoscopic mucosal resection (EMR), surgery alone, neo-adjuvant chemotherapy (NACT) or neo-adjuvant chemoradiotherapy (NACRT) prior to surgery, definitive chemo-radiotherapy (dCRT) or palliative therapy. The optimum treatment strategy was decided by consensus at the MDT. In general, fit patients with tumours pre-operatively staged as T3/T4a, N0/N1 were pre-operatively treated with NACT or NACRT. Less fit patients, or those with T1/2 N0 disease, had surgery alone. Patients deemed unsuitable for surgery due to co-morbidity and/or performance status, extensive loco-regional disease, or personal choice received dCRT.

### Data preparation and PET segmentation

Manual delineation of the metabolic tumour volume (MTV) is limited by intra- and inter-observer variability and is time consuming [17–19]. Semi-automated and automated segmentation methods are favourable alternatives by reducing variability in delineation and decreasing the contouring time [20]. Fixed percentage thresholding has been shown to be dependent upon the SUV_max_ of a tumour as well as the MTV [21]. Furthermore, it has been shown that texture analysis of PET imaging is dependent upon the segmentation method used to define the MTV [12, 22, 23]. However, more complex segmentation algorithms such as adaptive iterative thresholding (AT) have been shown to be independent of SUV_max_ as well as being correlated to the MTV. Segmentation methods adopting clustering techniques such as Fuzzy C-means (FCM), Gaussian fuzzy C-means (GCM) and K-means (KM) using 2, 3 and 4 clusters (FCM2, GCM3–4, KM2 - KM4), as well as region growing (RG) and watershed transform (WT) methods, are promising segmentation methods in the delineation of the MTV. These segmentation methods are reviewed in detail in the report by Hatt et al. [9], are described in detail previously [24] and are summarised in Table 1. In each case, the MTV was defined using AT, FCM2, GCM3, GCM4, KM2, KM3, KM4, RG and WT PET segmentation methods.

**Table 1:** Name and description of PET-AS methods used in this study, with references of published work using similar segmentation approaches

Algorithm	Description	Key references
AT	3D adaptive iterative thresholding, using background subtraction	Jentzen et al. [43], Drever et al. [44]
RG	3D region-growing with automatic seed finder and stopping criterion	Day et al. [45]
KM	3D K-mean iterative clustering with custom stopping criterion	Zaidi and El Naqa [8]
FCM	3D fuzzy C-mean iterative clustering with custom stopping criterion	Belhassen and Zaidi [46]
GCM	3D Gaussian mixture models based clustering with custom stopping criterion	Hatt et al. [37]
WT	Watershed transform-based algorithm, using sobel filter	Geets et al. [47], Tylski et al. [48]

A clinical radiologist subjectively assessed each tumour contour produced by all nine PET segmentation methods for accurate tumour representation. All tumour contours were visualised using the same software and image settings to ensure consistent methodology. Segmentation methods were considered inadequate for further analysis if less than 90% of contours were non-representative. This pre-defined value was decided upon prior to image visualisation. Contours were assessed individually and classified as not representative if contours were greatly different from the primary tumour, or included bone, lung or medistinial tissue. In addition, segmentation methods that had failed and conformed to the boundary of the bounding box were defined as not representative of the primary tumour.

### Clinical data and image analysis

Only primary tumours were analysed to ensure consistent methodology across all patients. Before quantitative image analysis and texture feature extraction, PET images were re-sampled into 0.5 SUV bins. A fixed bin width maintains a constant intensity resolution when compared to approaches based on a fixed number of bins [25]. In the development of the prognostic models, age at diagnosis (number of years), radiological stage (stage IA-IV) and treatment (curative vs palliative) were included because these are strong predictors of survival [26]. Curative and palliative treatments were coded as 1 and 2 respectively. Radiological staging was modelled categorically.

Radiomic analysis was performed using features implemented as part of the Image Biomarker Standardisation Initiative (IBSI), a multicentre, international collaboration aimed at improving the reproducibility and validation of quantitative medical image analysis studies [5]. The radiomic features selected for inclusion in this study were chosen as they have shown prognostic and predictive significance in other radiomic studies investigating OC [12, 27, 28]. These have been summarised in Table 2. Moreover, many radiomic feature implementations have been described [6, 7, 27, 29] and are divided into three groups for which a summary is provided. In this study, the MTV was analysed as a 3D volume with no thresholding applied to the MTV mask.

**Table 2:** Summary of quantitative imaging features

Type/order of statistics	Feature	Brief definition
Morphological	Volume	Sum of voxels delineated multiplied by the volume of one voxel
Pre-discretisation	SUV_max_	Maximum uptake of FDG in the MTV
	Energy	Sum squared SUV values in the MTV
First order	Skewness	Measures symmetry of intensity histogram
	Kurtosis	Measures flatness of intensity histogram
	Entropy	Measures randomness
Second order	Dissimilarity	Variation of grey level pairs (GLCM). Features were calculated for each unique direction and averaged with a distance setting of 1
Higher order	Grey-level non-uniformity	Distribution of zone counts for each intensity value (GLSZM)
	Zone percentage	Fraction of recorded zones compared to maximum possible
	Coarseness	Measures spatial rate of change in intensity using a distance of 1

### First-order metrics

First-order statistical metrics summarise the voxel intensity distribution within the segmented MTV, without concern for spatial relationships [30]. First-order metrics are typically histogram based and reduce the MTV to singular values describing the mean, minimum, maximum, median, and uniformity of the intensities within the MTV. Included in first-order stastical analysis is Skewness (asymmetry measure), Kurtosis (pointiness measure) and Entropy (randomness measure). Kurtosis and skewness have been shown to be independent predictors of survival [15] and of prognostic significance in the literature [31].

### Higher-order metrics

Higher-order statistical metrics retain spatial information and are used to quantify inter-voxel intensity relationships. Dissimilarity is the quantification of variation in voxel pairs and is calculated using a Grey Level Co-occurrence Matrix (GLCM) generated for each unique direction and averaged. A low dissimilarity is resultant of neighbouring voxels having similar values [32]. Zone percentage is calculated from a Grey Level Size Zone matrix (GLSZM) by assessing the fraction of recorded zones compared to the maximum number of possible zones. Heterogeneous MTVs have high zone percentage scores. Grey Level Non-Uniformity (GLNU) is an evaluation of the distribution of zone counts for each intensity value. The feature value is low when the number of zones associated with each intensity value are similar. Coarseness is a neighbourhood grey-tone difference matrix (NGTDM) feature that gives an indication of the level of spatial rate of change in intensity [33]. GLCM, GLSZM and NGTDM can be computed in 2D or 3D. The matrices in this study were computed in 3D as this may highlight the multi-scale, directional properties of tumour tissue [34].

### Outcome data

The primary outcome of the study was OS, defined as number of months survived from date of diagnosis. Patients were followed up 3-monthly for the first year, 6-monthly until 5 years then annually thereafter, or until death. All included patients were followed up for at least 12 months. Date of death was obtained from the Cancer Network Information System Cymru database (CaNISC, Velindre NHS Trust, Wales).

### Statistical analysis

Categorical variables were described as frequency (percent) and continuous variables as median (range) and differences assessed with appropriate non-parametric tests. Cumulative survival was calculated by the Kaplan-Meier life-table method. Cox regression models with backward conditional method were constructed using identical clinical data and imaging data derived from each of the segmentation methods. An individual prognostic score was calculated from each segmentation method by summation of the products of variables and their corresponding parameter estimate. Using this score, patients were separated into low, intermediate and high-risk groups (higher prognostic score deemed higher risk) and a log-rank test evaluated significant differences in OS. The number of patients that changed risk stratification group depending on the segmentation method used was calculated, and the OS for the different risk groups between models was analysed. A *p* value of < 0.05 was considered statistically significant. Statistical analysis was performed using SAS version 9.4 (SAS, NC, USA) and SPSS version 23.0 (IBM, Chicago, USA). Imaging data, software and delineated MTVs are not available publicly.

## Results

Four hundred and seventy-two patients, each with nine MTV contours delineated by AT, FCM2, GCM3, GCM4, KM2, KM3, KM4, RG and WT PET segmentation methods were assessed by a Clinical Radiologist with 5 years of research experience for accurate tumour representation. Forty-five patients and five segmentation methods were excluded due to poor MTV delineation. FCM2 failed to delineate an acceptable tumour representation in 145 (30.8%) of cases. KM3 and KM4 failed in 88 (18.6%) and 215 (45.6%) of cases, respectively. RG failed in 389 (82.5%), and GCM4 in 33 (7%) of cases. Therefore, 427 cases with MTVs delineated with KM2, GCM3, AT and WT PET segmentation methods deemed to have accurate tumour representation and included for further analysis.

The 427 cases included for analysis were used to develop the prognostic models for KM2, WT, GCM3 and AT methods. Baseline characteristics of patients are detailed in Table 3. The median OS of the cohort was 17.0 months (95% confidence interval (95% CI) 14.8–19.2). Median follow-up was 35.0 months (95% CI 28.7–41.3). Overall 1- and 2-year survival in the development cohort was 65.3% and 30.1%, respectively.

**Table 3:** Baseline characteristics of patient cohort

Median age	67.0 years (range 24–84)
Gender	Male 315 (73.8):female 112 (26.2)
Histology	
Adenocarcinoma	313 (73.3)
Squamous cell carcinoma	100 (23.4)
Undifferentiated	5 (1.2)
High-grade dysplasia	4 (0.9)
Neuro-endocrine	3 (0.7)
Small cell carcinoma	1 (0.2)
Sarcoma	1 (0.2)
Tumour location	
Oesophagus	268 (62.8)
Upper third	14 (5.2)
Middle third	71 (26.5)
Lower third	183 (68.3)
Gastro-oesophageal junction	159 (37.2)
Siewert I	67 (42.1)
Siewert II	42 (26.4)
Siewert III	50 (31.4)
Stage group	
IA	10 (2.3)
IB	17 (4.0)
IIA	70 (16.4)
IIB	13 (3.0)
IIIA	97 (22.7)
IIIB	52 (12.2)
IIIC	76 (17.8)
IV	92 (21.5)
Treatment	
Curative	224 (52.5)
NACT	86 (38.4)
dCRT	86 (38.4)
Surgery alone	31 (13.8)
NACRT	20 (8.9)
EMR	1 (0.5)
Palliative	203 (47.5)
Mortality	
Alive	132 (30.9)
Dead	295 (69.1)

### Development of prognostic models

The final steps of each prognostic model are presented in Table 4. Three known clinical prognostic factors (age, radiological stage and treatment) remained in each derived model, but there was a difference in the inclusion of texture metrics by segmentation technique. AT and KM2 produced the same model output. Interestingly, IBSI metrics were not included in the final models for these segmentation methods. However, skewness and kurtosis were independently significant for survival using GCM3 method. Skewness and GLNU were significant using WT method. Their inclusion in the models illustrates their additional prognostic value compared with current prognostic factors.

**Table 4:** Final output of prognostic models derived using AT, GCM3, KM2 and WT PET segmentation methods

	Parameter estimate	*p* value	Hazard ratio	95% CI
AT				
Age	0.020	0.001	1.020	1.008–1.033
Treatment	− 1.075	< 0.001	0.341	0.254–0.459
Stage	0.144	< 0.001	1.155	1.072–1.245
GCM3				
Age	0.019	0.003	1.019	1.006–1.032
Treatment	− 1.024	< 0.001	0.359	0.266–0.485
Stage	0.142	< 0.001	1.153	1.068–1.245
Kurtosis	0.632	0.002	1.882	1.260–2.809
Skewness	− 0.789	0.044	0.454	0.211–0.980
KM2				
Age	0.020	0.001	1.020	1.008–1.033
Treatment	− 1.075	< 0.001	0.341	0.254–0.459
Stage	0.144	< 0.001	1.155	1.072–1.245
WT				
Age	0.018	0.004	1.018	1.006–1.031
Treatment	− 1.063	< 0.001	0.345	0.257–0.464
Stage	0.140	< 0.001	1.150	1.065–1.242
GLNU	0.017	0.006	1.017	1.005–1.029
Skewness	0.674	0.030	1.962	1.067–3.607

### Prognostic score calculation

The equations for each model derived from different segmentation methods were used to calculate the prognostic scores, and are listed in Table 5. These calculations were derived using published methods [35].

**Table 5:** Prognostic model equations

Segmentation Method	Prognostic model equation
AT	(Age × 0.020 − (Treatment × 1.075) + (Stage × 0.144)
GCM3	(Age × 0.019) − (Treatment × 1.024) + (Stage × 0.142) − (Skewness × 0.789) + (Kurtosis × 0.632)
KM2	(Age × 0.020) − (Treatment × 1.075) + (Stage × 0.144)
WT	(Age × 0.018) − (Treatment × 1.063) + (Stage × 0.140) + (Skewness × 0.674) + (GLNU × 0.017)

**Fig. 1:**
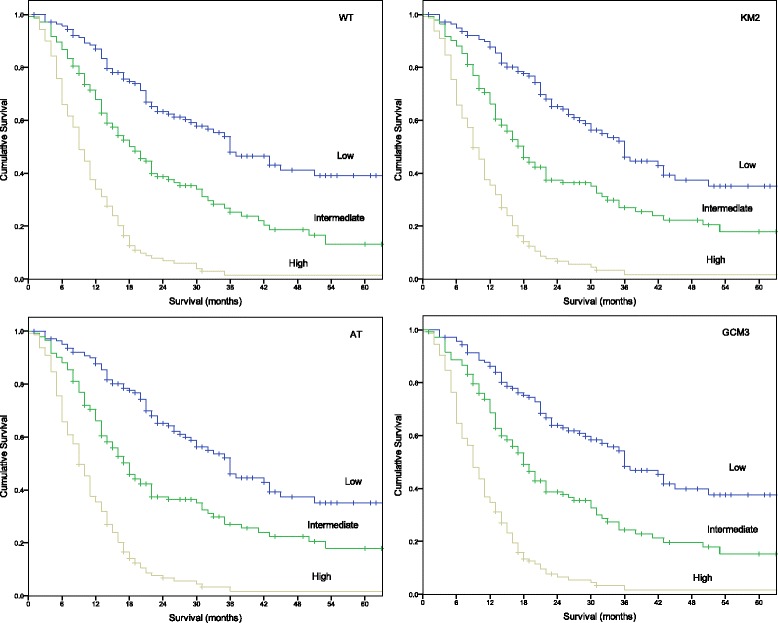
Risk stratification and OS for WT (top left), KM2 (top right), AT (bottom left) GCM3 (bottom right)

Figure 1 shows the risk stratification for WT, KM2, AT and GCM3. Median OS for the low-risk, intermediate-risk and high-risk groups in the AT- and KM2-derived prognostic model was 36.0 months (29.9–42.1 months), 18.0 months (15.1–20.9 months) and 9.0 months (7.8–10.2 months), respectively. Median OS for the low-risk, intermediate-risk and high-risk groups in the GCM3-derived prognostic model was 36.0 months (28.8–43.2 months), 18.0 months (15.4–20.6 months) and 9.0 months (7.7–19.2 months). Median OS for the WT derived prognostic model low-risk, intermediate-risk and high-risk groups was 36 months (27.8–44.2 months), 19 months (15.1–23 months) and OS for the high-risk group was 9 months (7.7–10.3 months) respectively. Table 6 shows the number of patients stratified as low, intermediate and high-risk for each single prognostic model along with the prognostic score range for each risk stratification group. Table 7 shows the number of patients whom change risk stratification.

**Table 6:** Number of patients in each risk stratification group for each single prognostic model and prognostic score range

Number of patients in risk group (prognostic range)	Low-risk	Intermediate-risk	High-risk
AT/KM2	141 (− 0.45–0.98)	143 (0.99–2.16)	143 (2.17–2.79)
GCM3	140 (− 1.13–0.36)	143 (0.37–1.54)	144 (1.55–2.73)
WT	142 (−0.17–1.30)	144 (1.31–2.48)	141 (2.49–3.62)

**Table 7:** Total number of patients and percentage that change risk-stratification group

Number changing group (%)	AT	GCM3	KM2	WT
AT				
GCM3	66 (15.4)			
KM2	0 (0.0)	66 (15.4)		
WT	57 (13.3)	73 (17.1)	57 (13.3)	

The largest proportion of patients to change risk stratification group was between prognostic models based on GCM3 and on WT (*n* = 73, 17.1%). It can be noted that no patient changed risk stratification group between AT and KM2 because the prognostic models were identical. The number of concordant patients stratified as low, intermediate and high-risk across the developed models was 118 (28%), 95 (22%) and 116 (27%), respectively. There was no overall survival difference between AT, GCM3, KM2 or WT low-risk groups (*χ*^2^ 0.052, df 3, *p* = 0.997), intermediate-risk groups (*χ*^2^ 0.016, df 3, *p* = 0.999) or high-risk groups (*χ*^2^ 0.028, df 3, *p* = 0.999).

For interest, Additional file 1 describes the developed prognostic models for the excluded PET-AS methods. Additional file 2 describes variances in radiomic features extracted using differing discretisation methodologies, which is an important consideration in radiomic studies. Additional file 3 describes the correlation of MTV with the extracted radiomic features.

## Discussion

Radiomic research aims to improve the prediction of patient outcome through the extraction of additional data from medical images. However, numerous challenges with the extraction of radiomic features have been highlighted [10]. Selection of significant features for prognostic models is of considerable importance because external parameters such as delineation method and image reconstruction parameters affect reproducibility and robustness of these features [14, 36].

In this study, first, second and higher-order radiomic features were extracted from each of the PET-AS delineations. The significant variables in the developed prognostic models were dependent upon the delineation method. In the GCM3-based prognostic models, first-order features kurtosis and skewness were found to be significant predictors of survival. However, higher-order feature GLNU was found to be of significance in combination with the first-order feature skewness in the WT-based prognostic model. For the AT and KM2-based prognostic models, radiomic features were not found to be significant predictors of survival in comparison to the currently known predictors such as clinical stage and age. This highlights the dependency of significant PET radiomic variables on segmentation method.

Our findings demonstrate the potential impact of different segmentation methods for prognostic models using standardised implementations of radiomic features within clinical practice. Patients may be assigned different risk stratification groups depending on the segmentation method used in the process of developing the prognostic model. This could lead to sub-groups of patients receiving a more aggressive treatment than is necessary, leading to decreased quality of life. Furthermore, patients could potentially be denied beneficial treatment.

Nine segmentation methods were included in this study, with five being excluded from analysis after being reviewed by a radiologist. These methods were excluded due to poor tumour representation in a number of cases. In Additional file 1, the prognostic models developed from PET-AS methods that were excluded from the study are described. Interestingly, the excluded PET-AS methods FCM2, KM3, KM4 and RG developed identical prognostic models to the included methods AT and KM2. This suggests that whilst radiomic features are dependent upon the delineation method, this may be unrelated to the delineation method considered acceptable by a radiologist.

It has been reported that the accuracy of the segmentation delineation of the MTV is dependent upon tumour characteristics [9, 21, 24]. GCM-based segmentation methods have been shown to have limited performance in low TBR scenarios [24]. Furthermore, clustering methods such as FCM are highly dependent upon the heterogeneity of the tumour volumes. In homogeneous regions with low TBRs, the iterative process of FCM has been shown to overestimate the tumour volume [37]. Statistical-based segmentation algorithms such as RG compare adjoining voxel intensities. If the voxels are of similar intensities, they are included within the volume [38]. However, the performance of statistical-based RG segmentation algorithms in highly heterogeneous tumour volumes is degraded. Moreover, the performance of RG is dependent upon the defined stopping criteria. In our study, the RG algorithm stopped voxel inclusion when after an iteration no more than 5% of the total number of voxels already defined as the MTV were included. This stopping criteria has been reported to be limited in complex tumours [24].

This study used radiomic data derived using SUV bins of 0.5 units. In Additional file 2, the variance of radiomic features derived using different discretisation methods is shown but lies outside the scope of this study, so further analysis was not performed. Future work could investigate how different discretisation methods influences the significance of radiomic features in the development of prognostic models and subsequent impact on risk stratification in patients with OC.

The variability in segmentation performance in any one single clinical case means the standardisation of the delineation of the MTV is critical for the application of radiomics within OC. This supports the recommendations of the International Atomic Energy Agency (IAEA) whom state that there are no validated quantitative approaches for PET contouring that will result in ideal tumour delineation for all patients and tumour locations [39]. In addition, the American Association of Physicist in Medicine (AAPM) Task Group No 211 reported that they could not recommend a single PET auto segmentation method for MTV delineation. However, machine-learned segmentation methods have showed promise for accurate MTV delineation [9]. Machine-learned-based and consensus-based segmentation methodologies have been proposed for the standardisation of the delineation of the MTV [20, 40, 41]. In Additional file 3, radiomic features derived from each segmentation method were correlated with MTV. As described, GLNU, Energy and Coarseness were correlated with MTV for all PET segmentation methods. However, the level of correlation varied between PET-AS methods. Our study suggests that a standardised segmentation methodology should be used for the development of prognostic models.

Shape metrics can also be quantified from the primary tumour. Within this group of radiomic features, the surface to volume ratio (S2VR), sphere to volume ratio, compactness, sphericity and disproportion of the tumour can be characterised but have not been included in this study which focused on intra-tumoural heterogeneity. However, studies have investigated the inclusion of shape metrics in prognostic models [42].

The results of this study are strengthened by the large cohort (*n* = 427) of OC patients with contours assessed and approved by a Clinical Radiologist. The approach of controlling model development by using identical clinical data and standardised image features ensured that differences in risk stratification were due to the image segmentation method. In this study, we did not use any PET image interpolation algorithm before image feature analysis [34]. This approach however, is consistent with currently reported studies.

## Conclusion

Prognostic models incorporating quantitative image features are dependent on the method used to delineate the primary tumour. This has a subsequent effect on risk stratification, with patients changing groups depending on the image segmentation method used. The standardisation of PET segmentation is important and should be considered in future prognostic and predictive clinical models. The findings of this study may have substantial potential impact on clinical management of patients with OC.

## Additional files

Additional file 1: Prognostic models developed from PET auto-segmentation methods excluded from the study. (DOCX 122 kb)

Additional file 2: Differences in radiomic features between two discretisation methods. (DOCX 69 kb)

Additional file 3: Correlation of radiomic features and the delineated Metabolic Tumour Volume. (DOCX 2375 kb)
