# Supplementary material for: Evaluation of prognostic models developed using standardised image features from different PET automated segmentation methods
Source: EJNMMI Res. 2018 Apr 11;8:29. doi: 10.1186/s13550-018-0379-3 (PMC5895559; doi:10.1186/s13550-018-0379-3)
Supplement: Supplementary file 1 — Prognostic models developed from PET auto-segmentation methods excluded from the study. (DOCX 122 kb) [file 13550_2018_379_MOESM1_ESM.docx]

Additional file 1

This document outlines prognostic models developed using segmentation methods Fuzzy clustering means with 2 clusters (FCM2), General clustering means with 4 clusters (GCM4), and k-means clustering with 3 and 4 clusters (KM3 and KM4) and region growing (RG). These segmentation methods were excluded from the main study because the assessing radiologist deemed the produced contour to not be representative in less than 90% cases. The final steps of each prognostic model are presented in Table 1. The equations for each model derived from different segmentation methods and that were used to calculate the prognostic scores are listed in Table 2. Overall survival (OS) and risk stratification for GCM4 is shown in Figure 1 and OS and risk stratification for FCM2, KM3, KM4 and RG is shown in Figure 2. Table 3 outlines the summary statistics of median OS (95% CI) for GCM4, FCM2, KM3, KM4 and RG and Table 4 shows the number of patients in each risk stratification group for each developed prognostic model. Table 5 shows the total number of patients and percentage that change risk-stratification group between each prognostic model.

Table 1: Final output of prognostic models derived using FCM2, GCM4, KM3, KM4 and RG PET-AS methods

| **FCM2** | Parameter Estimate | p-value | Hazard Ratio | 95% CI |
| --- | --- | --- | --- | --- |
| Age | 0.020 | 0.001 | 1.020 | 1.008 – 1.033 |
| Treatment | -1.075 | <0.001 | 0.341 | 0.254 – 0.459 |
| Stage | 0.144 | <0.001 | 1.155 | 1.072 – 1.245 |
|  |  |  |  |  |
| **GCM4** | Parameter Estimate | p-value | Hazard Ratio | 95% CI |
| Age | 0.020 | 0.001 | 1.021 | 1.008 – 1.033 |
| Treatment | -1.055 | <0.001 | 0.348 | 0.259 – 0.469 |
| Stage | 0.159 | <0.001 | 1.172 | 1.086 – 1.265 |
| Kurtosis | 0.207 | 0.016 | 1.230 | 1.039 – 1.455 |
|  |  |  |  |  |
| **KM3** | Parameter Estimate | p-value | Hazard Ratio | 95% CI |
| Age | 0.020 | 0.001 | 1.020 | 1.008 – 1.033 |
| Treatment | -1.075 | <0.001 | 0.341 | 0.254 – 0.459 |
| Stage | 0.144 | <0.001 | 1.155 | 1.072 – 1.245 |
|  |  |  |  |  |
| **KM4** | Parameter Estimate | p-value | Hazard Ratio | 95% CI |
| Age | 0.020 | 0.001 | 1.020 | 1.008 – 1.033 |
| Treatment | -1.075 | <0.001 | 0.341 | 0.254 – 0.459 |
| Stage | 0.144 | <0.001 | 1.155 | 1.072 – 1.245 |
|  |  |  |  |  |
| **RG** | Parameter Estimate | p-value | Hazard Ratio | 95% CI |
| Age | 0.020 | 0.001 | 1.020 | 1.008 – 1.033 |
| Treatment | -1.075 | <0.001 | 0.341 | 0.254 – 0.459 |
| Stage | 0.144 | <0.001 | 1.155 | 1.072 – 1.245 |
|  |  |  |  |  |

Table 2: Prognostic model equations

| **Segmentation Method** | **Prognostic Model Equation** |
| --- | --- |
| **FCM2** | (Age * 0.020) - (Treatment * 1.075) + (Stage * 0.144) |
| **GCM4** | (Age * 0.020) - (Treatment * 1.055) + (Stage * 0.159) + (Kurtosis * 0.207) |
| **KM3** | (Age * 0.020) - (Treatment * 1.075) + (Stage * 0.144) |
| **KM4** | (Age * 0.020) - (Treatment * 1.075) + (Stage * 0.144) |
| **RG** | (Age * 0.020) - (Treatment * 1.075) + (Stage * 0.144) |

Figure 1: Risk stratification and OS for GCM4

Figure 2: Risk stratification and OS for FCM2, KM3, KM4 and RG

Table 3: Summary statistics of median OS (95% CI) for GCM4, FCM2, KM3, KM4 and RG

|  |  |  | 95% Confidence Intervals | |
| --- | --- | --- | --- | --- |
| Segmentation Method | Risk Group | Median OS | Lower | Upper |
| GCM4 | Low | 36.000 | 27.393 | 44.607 |
|  | Intermediate | 18.000 | 14.554 | 21.446 |
|  | High | 9.000 | 7.704 | 10.296 |
| FCM2, KM3, KM4, RG | Low | 36.000 | 29.857 | 42.143 |
|  | Intermediate | 18.000 | 15.103 | 20.897 |
|  | High | 9.000 | 7.755 | 10.245 |

Table 4: Number of patients in each risk stratification group for GCM4, FCM2, KM3, KM4 and RG developed prognostic models

| number of patients in risk group  (prognostic range) | Low Risk | Intermediate Risk | High Risk |
| --- | --- | --- | --- |
| FCM2 / KM3 / KM4 / RG | 141  (-0.45 – 0.98) | 143  (0.99 – 2.16) | 143  (2.17 – 2.79) |
| GCM4 | 141  (-0.13 – 1.02) | 142  (1.03 – 2.19) | 144  (2.20 – 3.14) |

Table 5: The total number of patients and percentage that change risk-stratification group

| Number changing group  (%) | AT/FCM2/KM2/  KM3/KM4/RG | GCM3 | GCM4 | WT |
| --- | --- | --- | --- | --- |
| AT/FCM2/KM2/  KM3/KM4/RG |  |  |  |  |
| GCM3 | 66  (15.4) |  |  |  |
| GCM4 | 51  (11.9) | 65  (15.2) |  |  |
| WT | 57  (13.3) | 73  (17.1) | 68  (15.9) |  |
