# Supplementary material for: Evaluation of prognostic models developed using standardised image features from different PET automated segmentation methods
Source: EJNMMI Res. 2018 Apr 11;8:29. doi: 10.1186/s13550-018-0379-3 (PMC5895559; doi:10.1186/s13550-018-0379-3)
Supplement: Supplementary file 2 — Differences in radiomic features between two discretisation methods. (DOCX 69 kb) [file 13550_2018_379_MOESM2_ESM.docx]

Additional file 2

This document compares differences in the radiomic features between two different discretisation methods. The two discretisation methods are discretization to 64 bins and a fixed bin with of 0.5 SUV units. Figures 1 – 10 show the differences in the median, minimum and maximum value of the extracted radiomic features. Variances in radiomic feature extraction may lead to changes in patient risk stratification, but this has not been tested in this study.

Figure 1: Median, min and max coarseness values for each PET-AS method extracted using 0.5 and 64 fixed binning methods

Figure 2: Median, min and max dissimilarity values for each PET-AS method extracted using 0.5 and 64 fixed binning methods

Figure 3: Median, min and max energy values for each PET-AS method extracted using 0.5 and 64 fixed binning methods

Figure 4: Median, min and max entropy values for each PET-AS method extracted using 0.5 and 64 fixed binning methods

Figure 5: Median, min and max entropy values for each PET-AS method extracted using 0.5 and 64 fixed binning methods

Figure 6: Median, min and max kurtosis values for each PET-AS method extracted using 0.5 and 64 fixed binning methods

Figure 7: Median, min and max, maximum grey level for each PET-AS method extracted using 0.5 and 64 fixed binning methods

Figure 8: Median, min and max, maximum grey level for each PET-AS method extracted using 0.5 and 64 fixed binning methods

Figure 9: Median, min and max Skewness for each PET-AS method extracted using 0.5 and 64 fixed binning methods

Figure 10: Median, min and max zone percentage for each PET-AS method extracted using 0.5 and fixed binning methods
