# Supplementary material for: Evaluation of prognostic models developed using standardised image features from different PET automated segmentation methods
Source: EJNMMI Res. 2018 Apr 11;8:29. doi: 10.1186/s13550-018-0379-3 (PMC5895559; doi:10.1186/s13550-018-0379-3)
Supplement: Supplementary file 3 — Correlation of radiomic features and the delineated Metabolic Tumour Volume. (DOCX 2375 kb) [file 13550_2018_379_MOESM3_ESM.docx]

Additional file 3

This document outlines the correlation of the radiomic features considered significant within the developed prognostic models and the delineated Metabolic Tumour Volume (MTV) in mL. Figures 1 – 9 show the correlation of extracted radiomic features with the MTV.


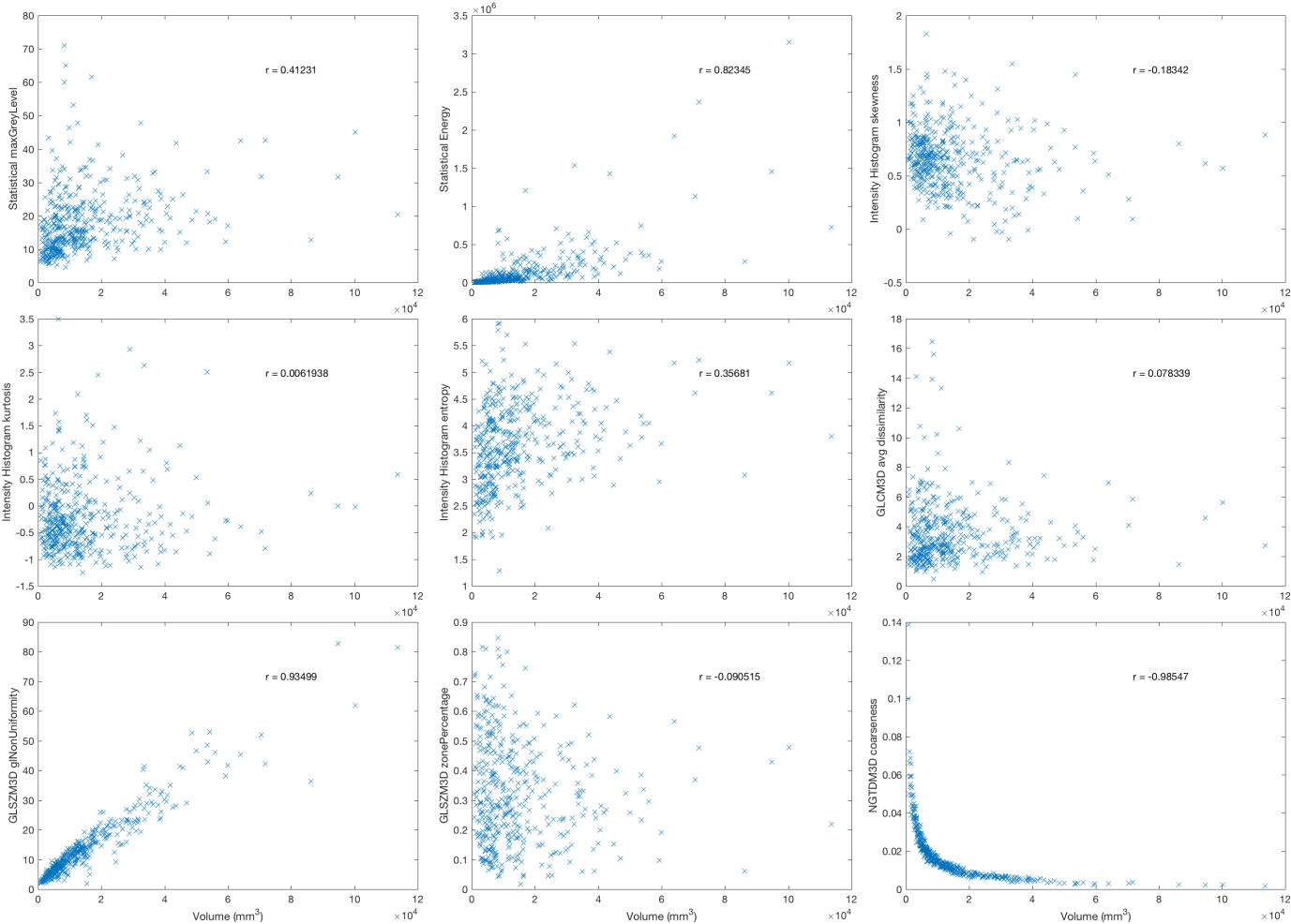


Figure 1: Correlation of radiomic features extracted from the AT PET-AS method with the Metabolic Tumour Volume derived by AT


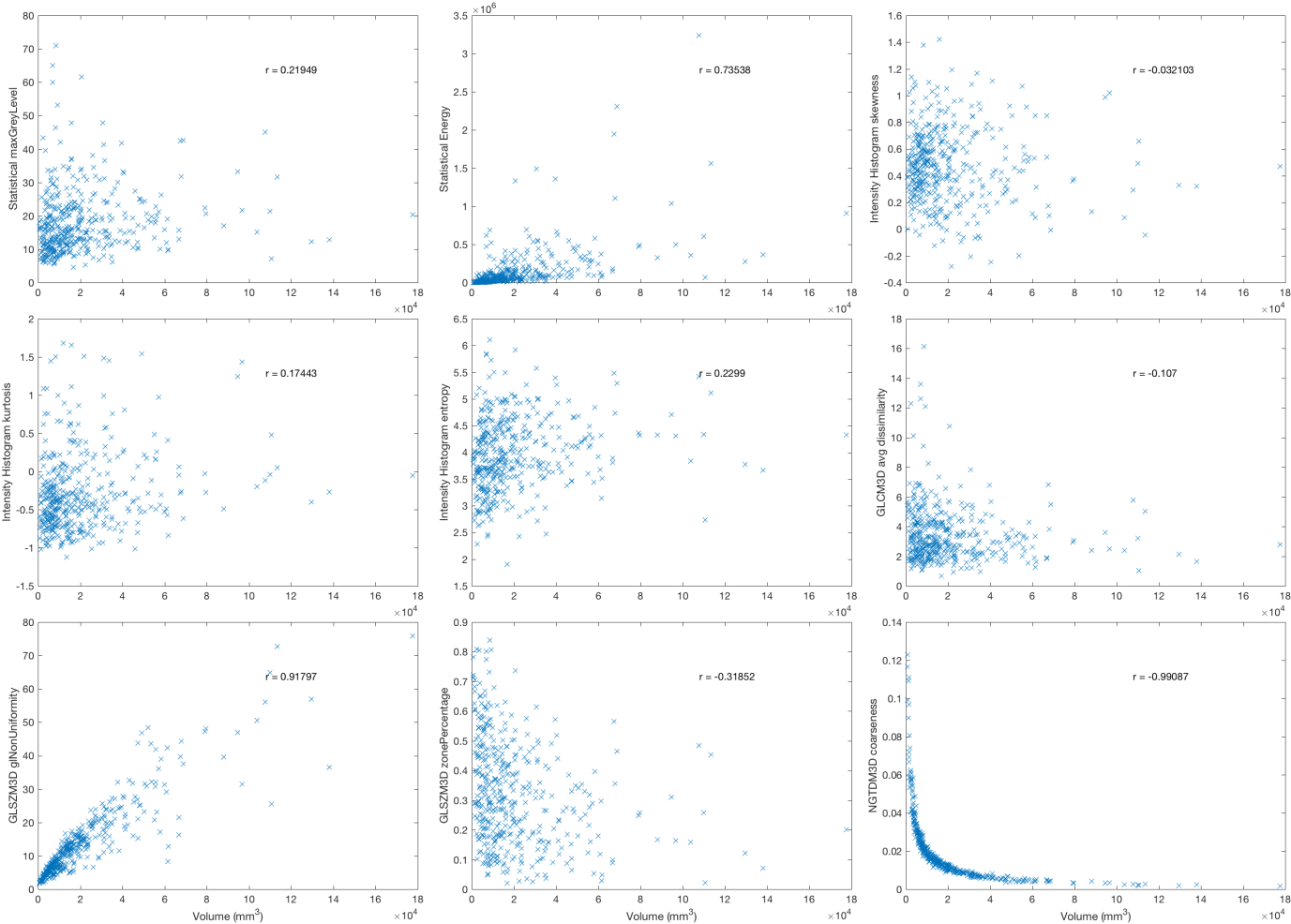


Figure 2: Correlation of radiomic features extracted from the GCM3 PET-AS method with the Metabolic Tumour Volume derived by GCM3


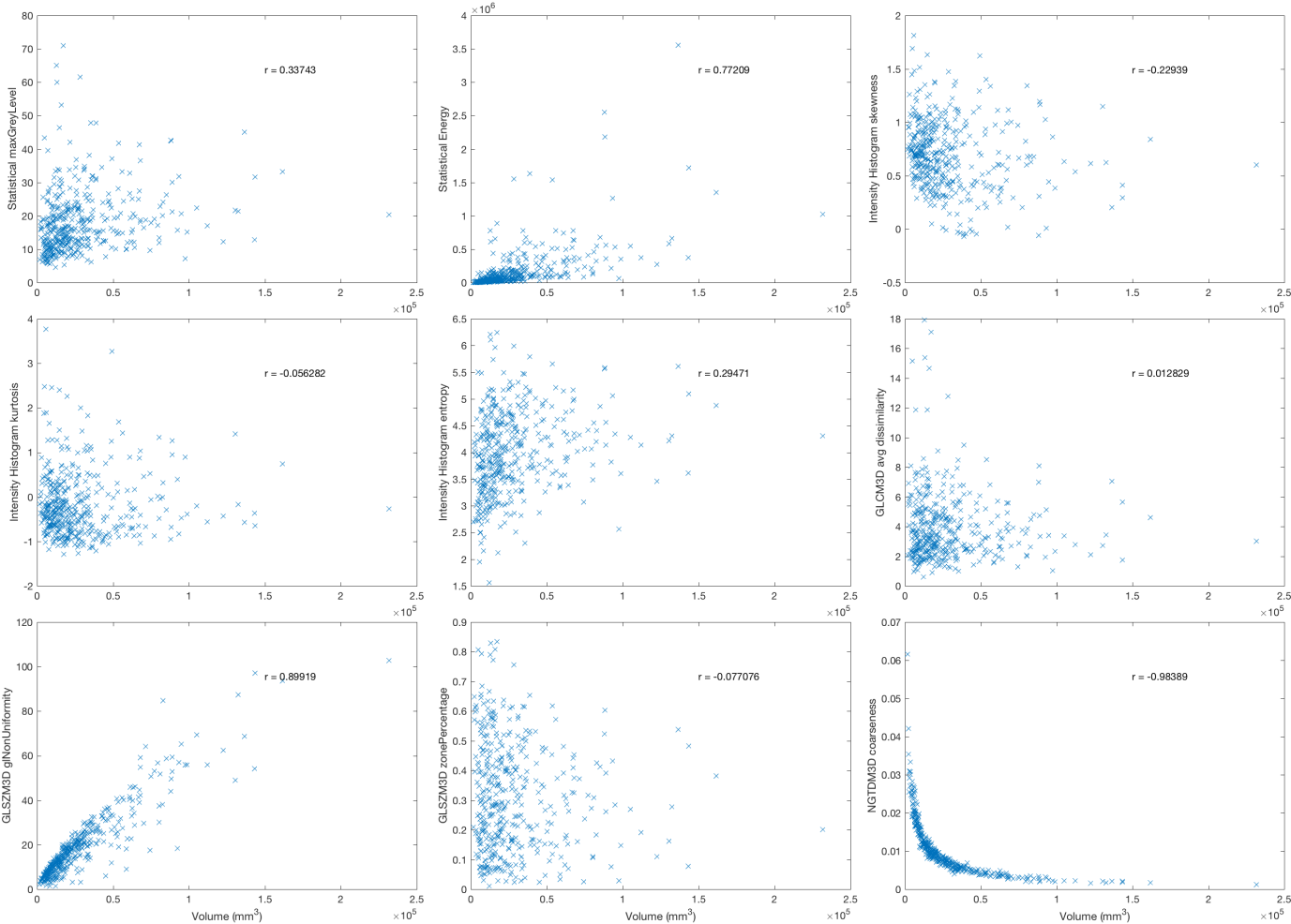


Figure 3: Correlation of radiomic features extracted from the KM2 PET-AS method with the Metabolic Tumour Volume derived by KM2


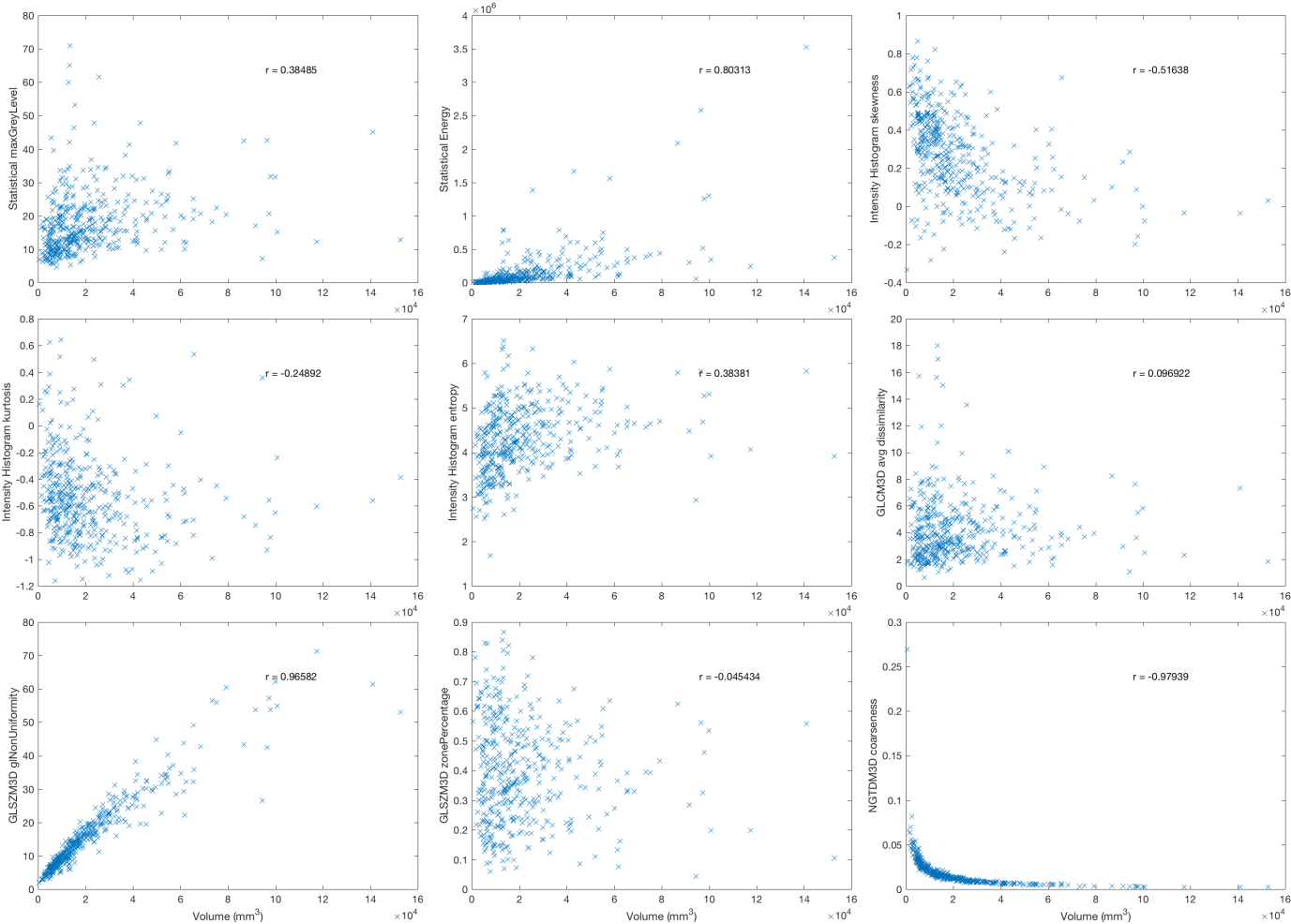


Figure 4: Correlation of radiomic features extracted from the WT PET-AS method with the Metabolic Tumour Volume derived by WT


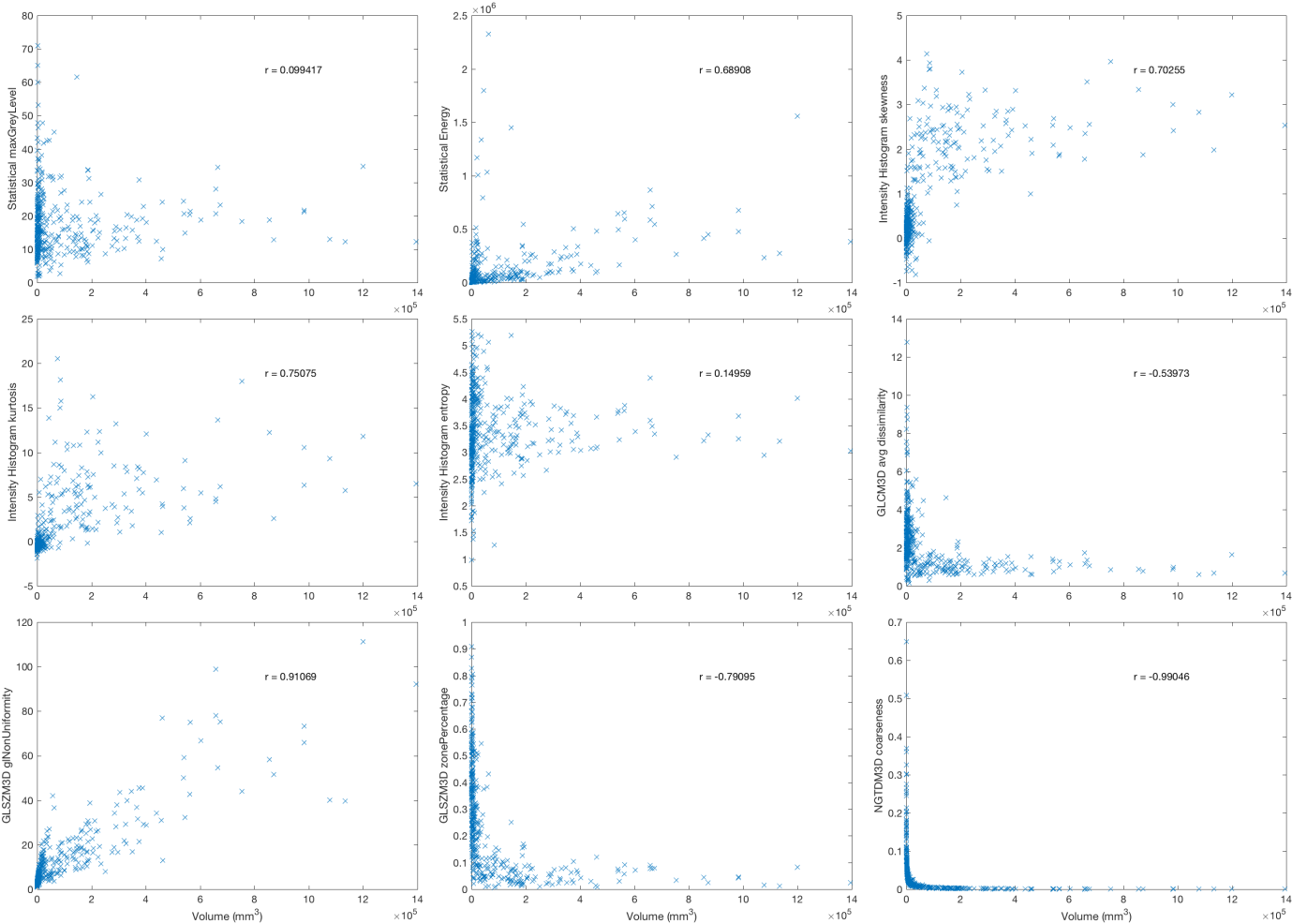


Figure 5: Correlation of radiomic features extracted from the FCM2 PET-AS method with the Metabolic Tumour Volume derived by FCM2


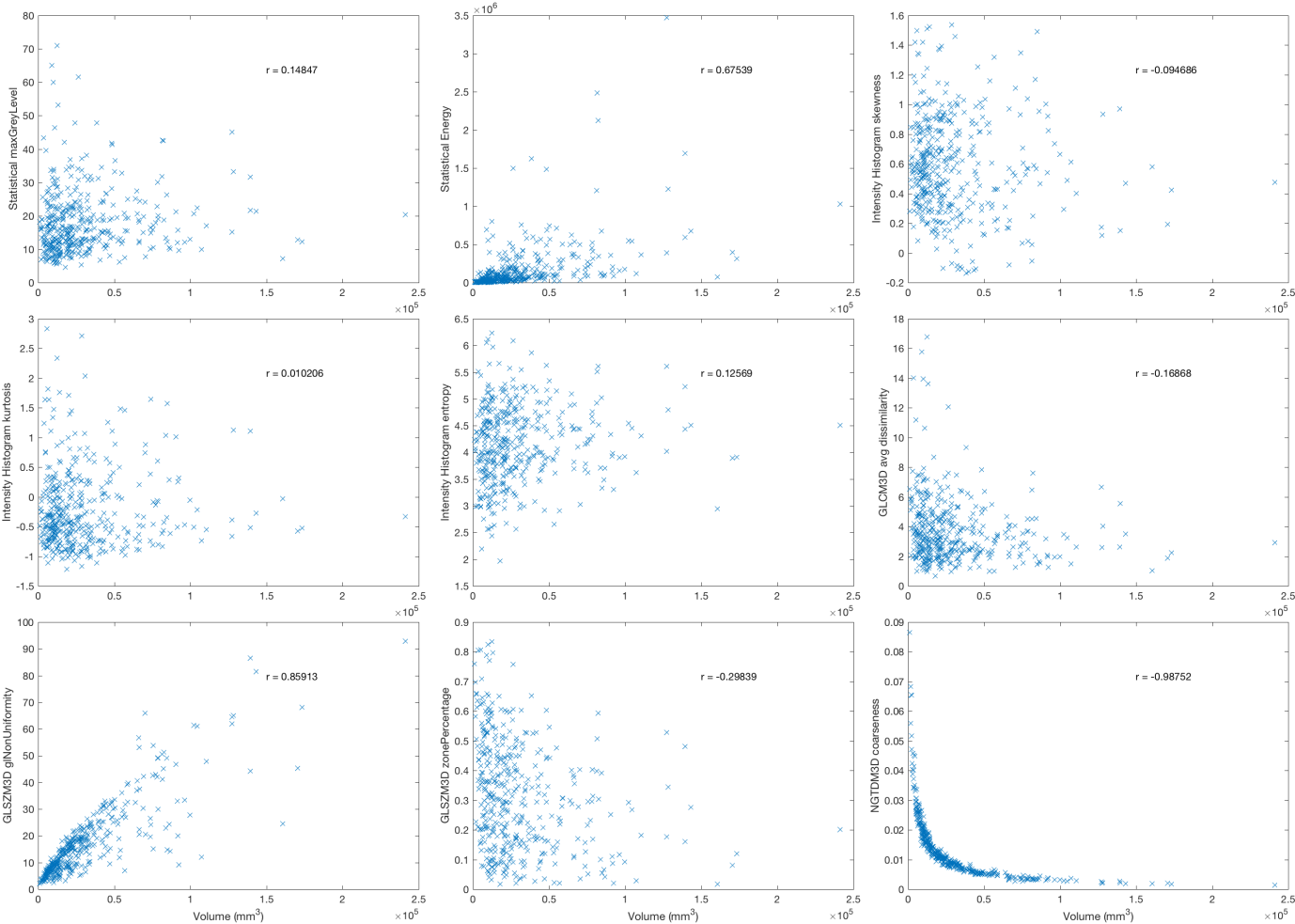


Figure 6: Correlation of radiomic features extracted from the GCM4 PET-AS method with the Metabolic Tumour Volume derived by GCM4


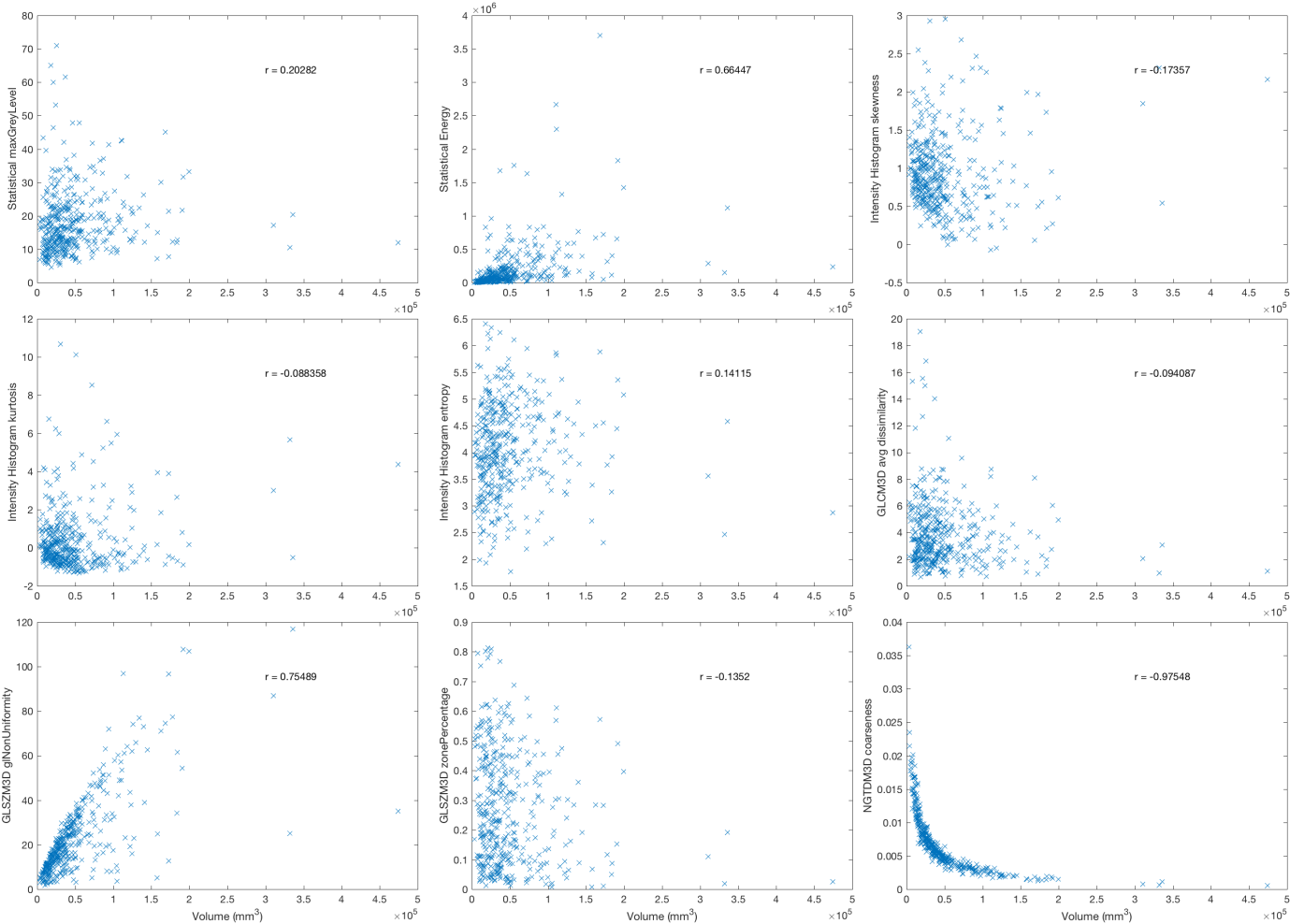


Figure 7: Correlation of radiomic features extracted from the KM3 PET-AS method with the Metabolic Tumour Volume derived by KM3


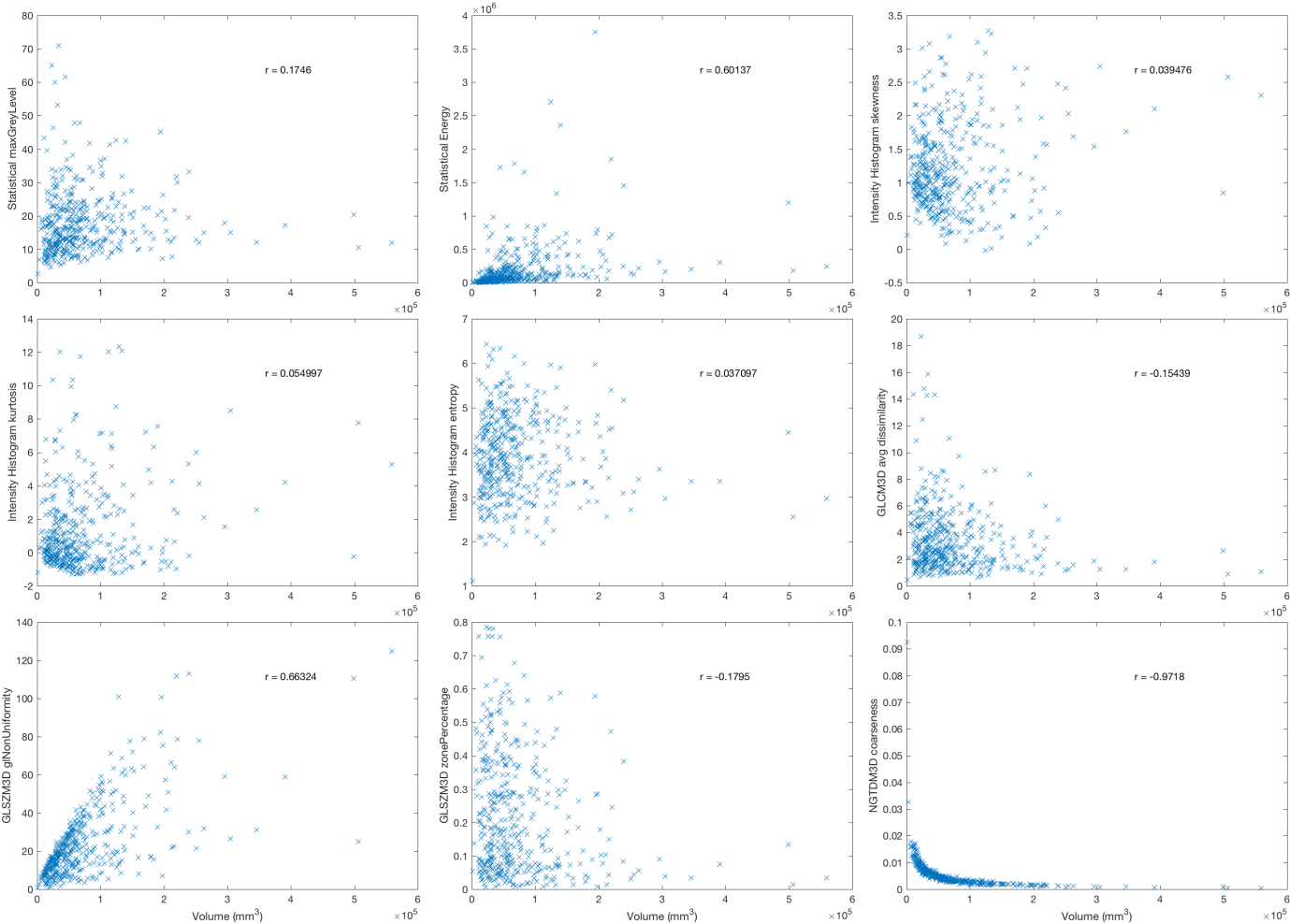


Figure 8: Correlation of radiomic features extracted from the KM4 PET-AS method with the Metabolic Tumour Volume derived by KM4


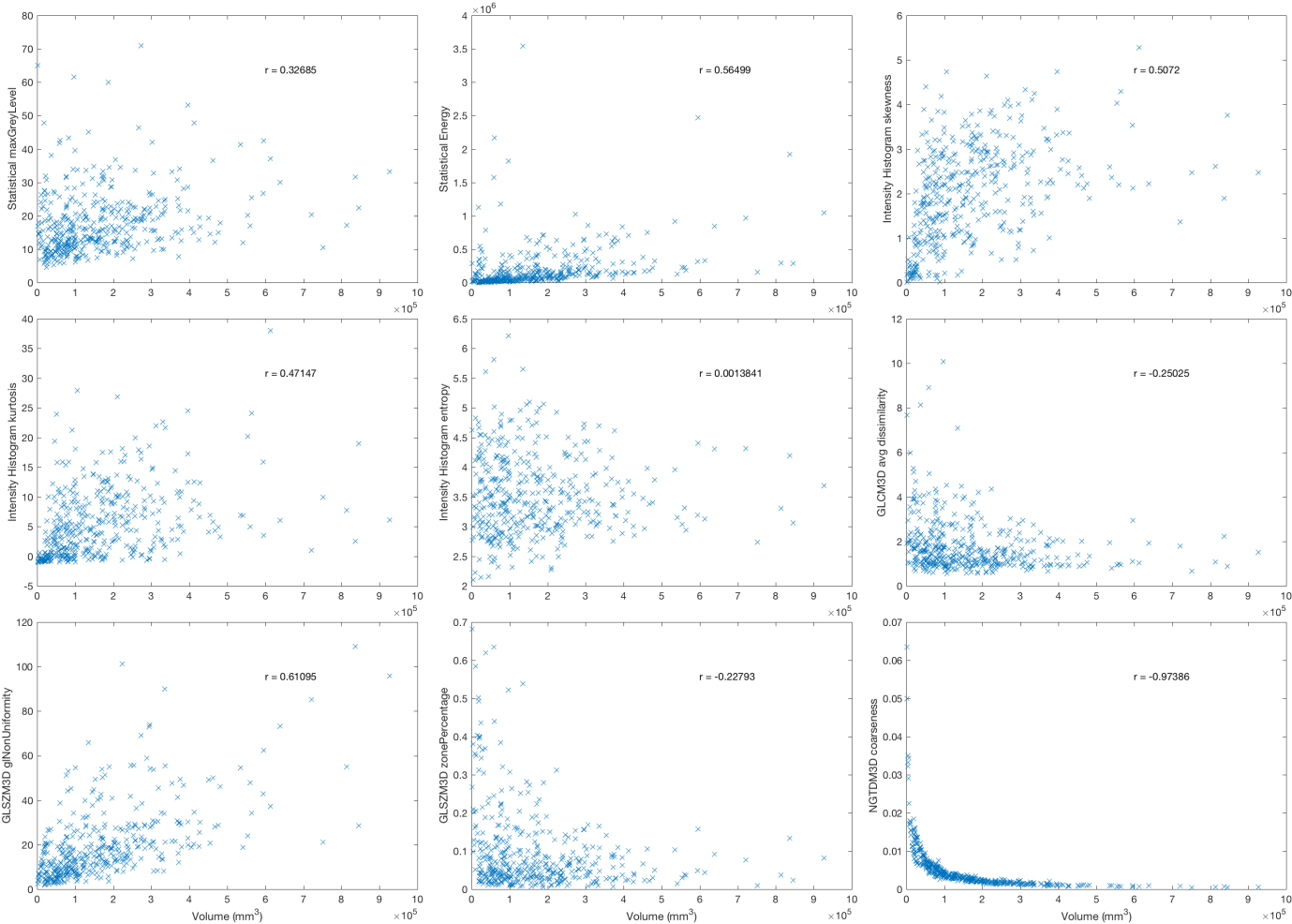


Figure 9: Correlation of radiomic features extracted from the RG PET-AS method with the Metabolic Tumour Volume derived by RG
